# Optical intensity changes under static load and their dependence on dental implant design around bone-mimicking material

**DOI:** 10.1117/1.JBO.30.11.115003

**Published:** 2025-11-18

**Authors:** Martynas Vencius, Pijus Beleckas, Paulius Kantakevičius, Julius Vengelis, Jan Pavel Rokicki, Dainius Razukevičius, Gintaras Janužis

**Affiliations:** a Lithuanian University of Health Sciences, Medical Academy, Faculty of Odontology, Kaunas, Lithuania; b Vilnius University, Laser Research Center, Vilnius, Lithuania; c Lithuanian University of Health Sciences, Department of Maxillofacial Surgery, Kaunas, Lithuania

**Keywords:** dental implants, bone mimicking material, photoelastic analysis, diameter, length

## Abstract

**Significance:**

Dental implants (DI) are among the most effective solutions for restoring masticatory function in patients with tooth loss. The success of these implants often depends on selecting appropriate design parameters, such as length and diameter, to ensure optimal outcomes. Understanding how these variables influence load transfer to the surrounding bone is essential for improving DI performance.

**Aim:**

We aimed to evaluate the effects of implant diameter and length on static load distribution to surrounding bone-mimicking material (BMM) under identical optical and mechanical conditions, using an original and more accurate photoelastic testing method.

**Approach:**

Epoxy resin was used to replicate the mechanical behavior of the bone under static load conditions. A total of 12 DI designs with varying lengths and diameters were tested, with three replicas each (n=36). Polarized light was applied to the apex of each implant to detect optical intensity changes (ΔI) in the BMM under a 150-N static load and at rest.

**Results:**

A significant correlation was found between implant diameter and load distribution (p<0.05). Wider implants showed more uniform load transfer, with 4.5-mm versus 5.5-mm-diameter implants showing 2.47 times less polarized light change, and 4.5-mm versus 6.9-mm implants showing 18.38 times less. By contrast, implant length had no statistically significant impact on load distribution (p>0.05). The 6.9-mm diameter and longest implants transmitted the highest load to the BMM, whereas 11.5-mm length implants showed the lowest optical intensity changes (ΔI) under static load.

**Conclusions:**

Implant diameter has a greater impact than length on stress distribution to surrounding structures. Emphasizing diameter selection may enhance implant longevity and clinical success.

## 1 Introduction

Modern approaches to restoring masticatory function offer a wide array of solutions, with titanium dental implants (DI) emerging as one of the most reliable and effective options. Introduced in 1965,^1^ titanium DI has since become a cornerstone in dental rehabilitation.

Current testing methods for titanium DI typically focus on parameters such as static load resistance,^2^ biological compatibility,^3^ maximum torque and fracture angles,^4^ and the intrinsic material properties of titanium.^5^ These tests are typically conducted by manufacturers or independent evaluators to ensure the structural integrity and quality of the DI. By contrast, clinical testing methods performed by practitioners are generally focused on assessing biological responses—particularly osseointegration and primary stability—rather than the mechanical properties of the DI itself. Among these, resonance frequency analysis (RFA) is increasingly utilized in clinical practice during surgical procedures to monitor DI stability in real-time.^6^^,^^7^

Despite innovative testing methods for DI, the primary stability can be effectively controlled by appropriately selecting DI diameter and length in specific clinical scenarios. In addition, high masticatory forces frequently pose challenges to DI stability, particularly if DI dimensions are inadequately chosen.^8^ Comparative analyses exploring the impact of varying DI diameters and lengths are extensively documented. Among mathematical modeling techniques used to assess DI stability, finite element analysis (FEA) has emerged as one of the solutions.^9^ Studies employing FEA have indicated that narrower DI typically experience smaller overload zones compared with wider DI. Conversely, shorter DI tend to exhibit lower stress levels than their longer counterparts.^10^^,^^11^ Although DI diameter and length are critical factors influencing load distribution, the thread design also significantly affects stress dispersion. Square-threaded DI designs have been consistently reported to reduce stress concentrations both within the DI body and in the adjacent bone structures.^12^^,^^13^ Furthermore, integrating curved flanks into buttress and reverse-buttress thread geometries has demonstrated additional reductions in stress distribution, enhancing overall DI performance.^12^ In addition, dual-surface DI featuring a combination of polished and porous-beaded regions has been shown to facilitate more favorable stress distribution to the surrounding bone tissue.^14^

On the contrary, FEA relies on theoretical assumptions and idealized boundary conditions, which may not fully represent the complexities of real-world biomechanical behavior. By contrast, experimental photoelastic methods enable direct, real-time visualization of stress distribution in a physical bone-mimicking material (BMM) under standardized static loading. Due to its capacity to provide tangible and measurable results, the photoelastic approach can serve as a valuable validation tool to complement and confirm FEA simulations.^15^

This innovative method enables a more precise assessment of the interaction between DI and bone by evaluating the static load transferred from the DI to the BMM. The approach incorporates laser-based investigations, offering high sensitivity and precision across various DI regions. By adjusting the laser beam width, specific points within the BMM or the entire area surrounding the DI can be assessed. Unlike traditional photoelastic analysis, which typically uses color gradient visualization, this study presents quantitative numerical data based on pixel-level intensity variations captured by a charge-coupled device (CCD) camera, thereby providing a more objective evaluation.^15^^,^^16^ Its methodological refinement improves reproducibility, precision, and comparability across different DI designs and their mechanical interactions with BMM under standardized loading conditions, in accordance with established photoelastic methodologies.^17^

This research offers valuable insights into the reliability of DI under clinically relevant conditions, emphasizing the role of DI diameter and length in load distribution. The findings hold potential to guide the optimization of DI design, enhancing their durability and functional stability. Eventually, the objective of our study was to analyze how polarized light optical intensity changes (ΔI) under static load are influenced by the DI design parameters, specifically their diameter and length.

Recent work has brought digital photoelasticity into DI biomechanics, including all-on-four configurations and 3D modeling, and has benchmarked photoelastic readouts against digital image correlation (DIC) and FEA for comparative evaluation of load transfer.^18^^,^^19^ This literature supports the use of optical metrics (ΔI or fringe behavior) to rank geometries under controlled conditions, which is the focus of our study.

## 2 Materials and Methods

### 2.1 Testing and Selection of BMM

To evaluate the mechanical stability and optical response of different BMM, three chemically curable epoxy resin formulations from the brand *Resin PRO*—“Transparente,” “FL210,” and “Liquidissima”—were tested without embedded DI. Each BMM type was cast into transparent rectangular molds measuring 10  mm×10  mm×30  mm, fabricated using a 3D printer. The resins were dispensed into the molds using disposable pipette tips. To prevent air bubble formation, a vacuum chamber was used immediately after pouring. The filled molds were then left undisturbed for 1 week to allow complete chemical curing. The bottom of each workpiece was rounded to allow proper insertion into a custom-made press for testing.

Three samples were prepared for each BMM type (n=3 per material). A compressive force of 150 N was applied gradually at a rate of 30 N/s with a custom-made press and weight controller. The optical response was assessed by measuring changes in light intensity under load, comparing values at 150 N with baseline values at 0 N. Visual recordings were captured at two positions on each workpiece—2 mm and 15 mm from the top, centered in the middle of the workpiece—and consistency between these recordings was evaluated.

The coefficient of determination ($R^{2}$) was used as the primary metric to assess signal reproducibility and material consistency. Among the tested resins, “Transparente” demonstrated the highest $R^{2}$ values, indicating superior stability and reliability under mechanical loading. As a result, “Transparente” was selected for use in all subsequent DI casting procedures.

### 2.2 Casting and Preparation of DI in BMM

Twelve different DIs from the UFII system by DIO Corporation (Busan, South Korea) were used in this study. Each DI type was cast into the chemically curable BMM “Transparente” with three replicates per type (total n=36), under standardized conditions. The DI were categorized by diameter and length as follows:

• 3.0-mm diameter with lengths of 8.5 mm (3085), 11.5 mm (30,115), and 15 mm (30,150)
• 4.5-mm diameter with lengths of 8.5 mm (4585), 11.5 mm (45,115), and 15 mm (45,150)
• 5.5-mm diameter with lengths of 8.5 mm (5585), 11.5 mm (55,115), and 15 mm (55,150)
• 6.9-mm diameter with lengths of 8.5 mm (6985), 11.5 mm (69,115), and 15 mm (69,150)

**Fig. 1:**
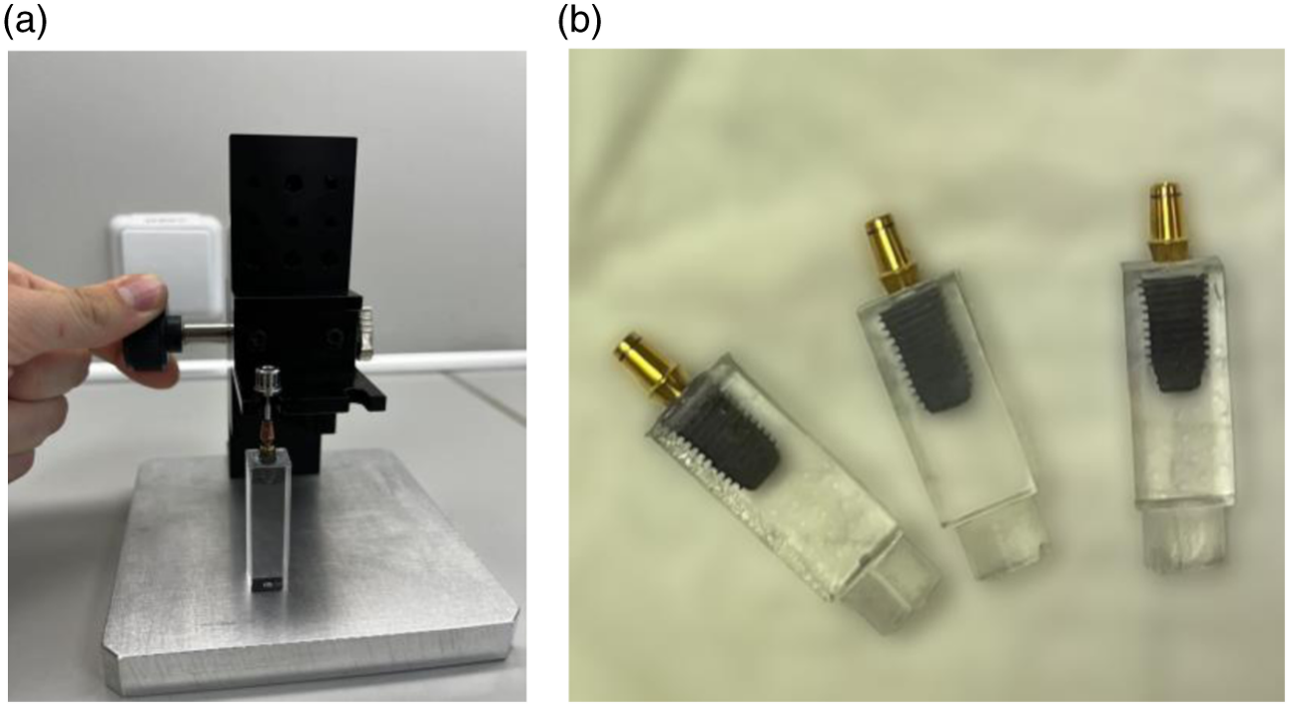
(a) Custom-made stand and 3D printer molds for DI casting into BMM. (b) Fully hardened BMM with cast-in DI.

At the beginning of the casting process, straight anatomical abutments were screwed onto the DI using a DI screwdriver and placed into a custom-made stand. DI were then lowered into molds measuring 10  mm×10  mm×30  mm, fabricated using a 3D printer [Fig. 1(a)]. The molds were filled with BMM using disposable pipette tips. To prevent air bubble formation, a vacuum chamber was used. After filling, the workpieces were left undisturbed for 1 week to allow complete polymerization and chemical curing. Once fully hardened, the BMM casts were removed from the molds and cleaned using steam (*Steamer X3*). The bottom of each workpiece was rounded to allow proper insertion into a custom-made press for testing [Fig. 1(b)].

### 2.3 Experimental Setup

**Fig. 2:**
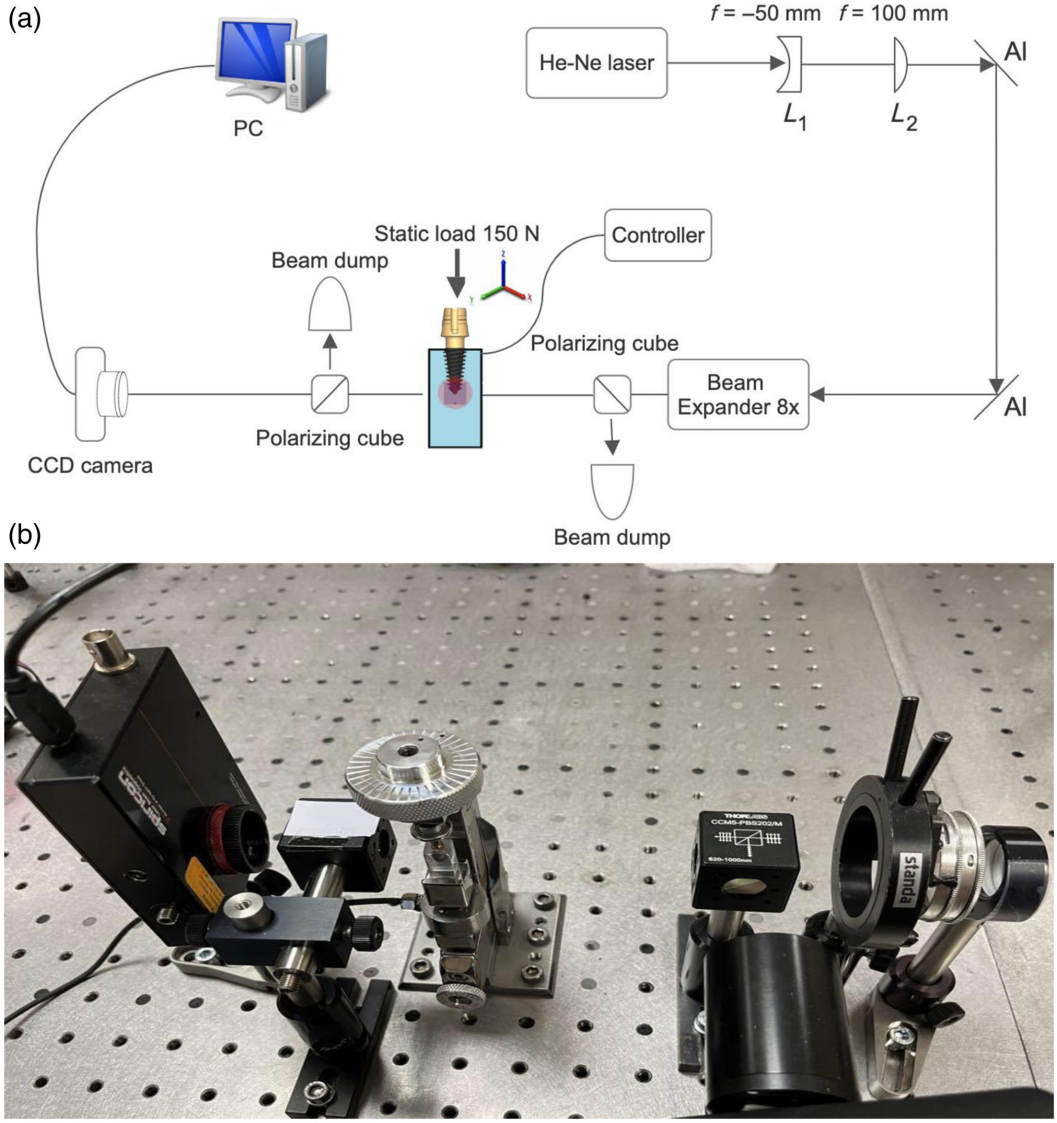
(a) Polarized light setup for DI apical area static load recordings. (b) The main devices that were used to detect changes in polarized light passing through the BMM.

A He-Ne laser (*model: HN-2P*, *VM-TIM GmbH*) served as the primary light source, emitting a coherent beam directed through a series of optical components to generate and analyze polarized light. Initially, the laser beam passed through two lenses (L1 with a focal length of −50  mm and L2 with a focal length of +100  mm), which adjusted the beam’s divergence and convergence. The beam was then reflected using aluminum mirrors (Al) and directed into a tunable beam expander (*Optogama VEX18*, *VEX series*). The expander increased the beam diameter to ensure it covered the full apical region of the DI. Next, the expanded beam passed through a locally monotonic polarizer, allowing only light with a specific polarization orientation to continue. This locally monotonic polarized light then entered the first polarizing cube, which further refined the polarization. The polarized beam was subsequently directed into a custom-designed mechanical press, where the prepared samples—each consisting of a DI embedded in BMM—were positioned. A vertical static load of 150 N was applied gradually—30 N/s to the DI through the custom-made press and weight controller. The sample was aligned such that the laser beam passed directly through the apical area of the DI and the surrounding BMM. After passing through the sample, the beam encountered a second polarizing cube (analyzer), oriented to transmit only light polarized perpendicularly to the initial polarizer. Finally, the transmitted polarized light was captured using a CCD camera (*model: SP602U*). In addition, two beam dumps were placed at strategic points in the setup to absorb stray light and prevent interference. The captured light optical intensity changes (ΔI) under static load were visualized and recorded using BeamStar 1.52 software on a connected personal computer (*PC*) [Figs. 2(a) and 2(b)].

### 2.4 Recording of Static Load Distribution on DI in a BMM

**Fig. 3:**
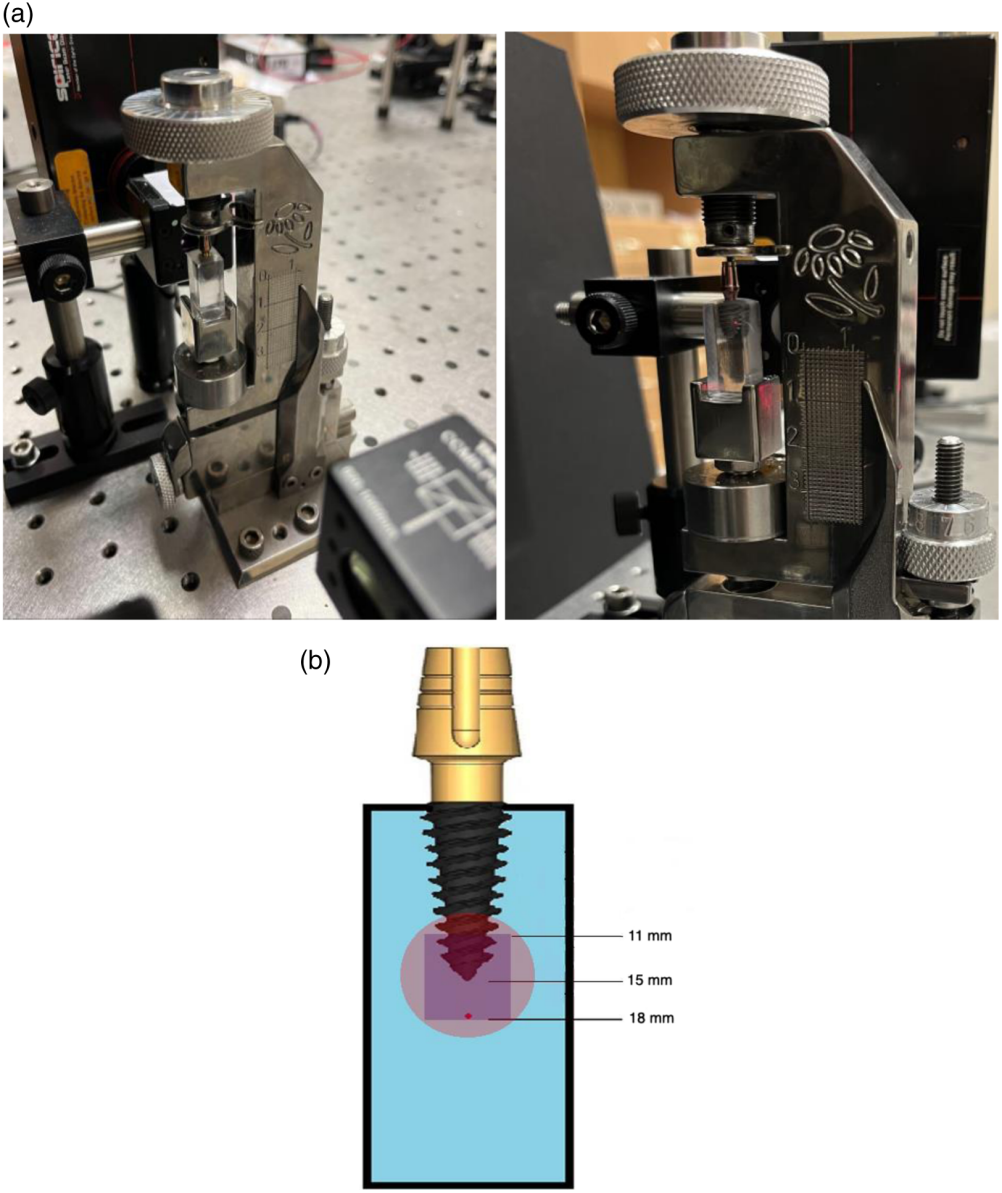
(a) Custom-made press for DI static load recordings with a sample of the inserted DI during the recordings. (b) Schematic representation of the 15-mm length DI in the BMM sample. Examining the DI apex with polarized light. Red color—polarized light—purple color—camera view—were recorded, covering 7 mm of vertical view: 3 mm of the DI and 4 mm of the BMM under the DI.

**Fig. 4:**
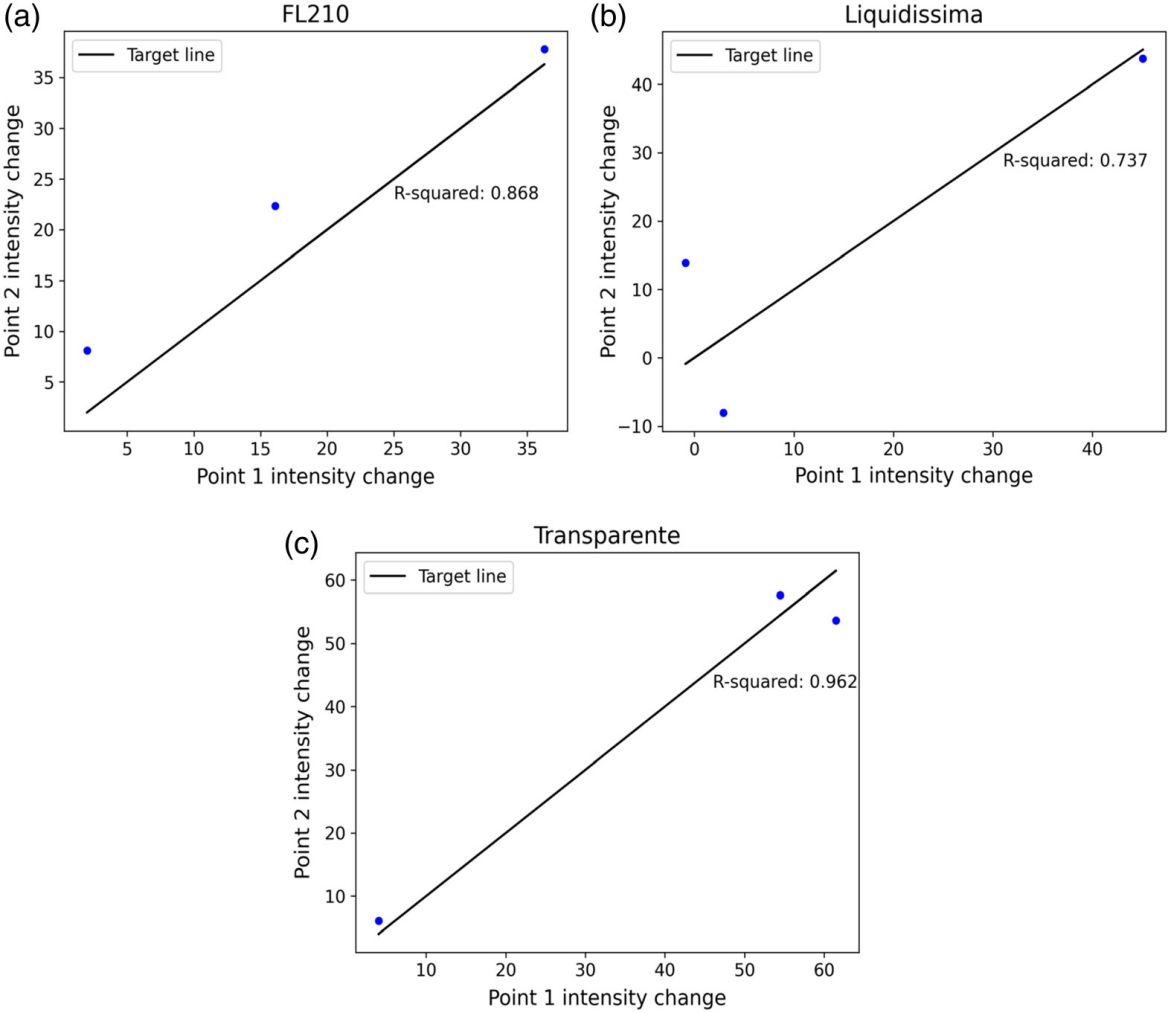
Example of 3085 DI third replica under 150 N static load: polarized light changes 2D view, standardized values.

The optical intensity changes (ΔI) under static load of polarized light were recorded using a CCD camera (SP602U) and BeamStar 1.52 software. A sample with a straight abutment was positioned in a custom-made static load press [Fig. 3(a)]. Polarized light was focused on the apical area of the DI, with 4 mm of the apical DI and 3 mm of BMM under, as shown in the schematic representation of the sample [Fig. 3(b)]. Initial recordings were taken with the sample under no load (0 N). Subsequently, a compressive force of 150 N was applied, and the changes in polarized light intensity in the apical area of the DI were recorded (Fig. 4). Polarizer and analyzer were crossed; analyzer angle was set on the high-sensitivity slope of the intensity–retardation curve to maintain a locally monotonic response across the region of interest (ROI). Exposure and dynamic range were verified to avoid clipping; ΔI was computed as the pixel-wise change between 0 N and 150 N for (i) the full frame and (ii) a predefined 3-mm apical ROI. Under these conditions, the field remained low-order; no multi-fringe patterns or phase wrapping were observed.

### 2.5 Statistics

To understand how the intensity of the sample changed under different loads, the percentage change was calculated between the unloaded condition (0 N) and the loaded condition (150 N). This was done using the formula [Eq. (1)].

$$\text{Optical intensity changes}\;(\Delta I)\;\text{under static load}=\frac{\text{Intensity at}\;150\;N-\text{intensity at}\;0\;N}{\text{Intensity at}\;0\;N}\times 100.$$ (1)

**Fig. 5:**
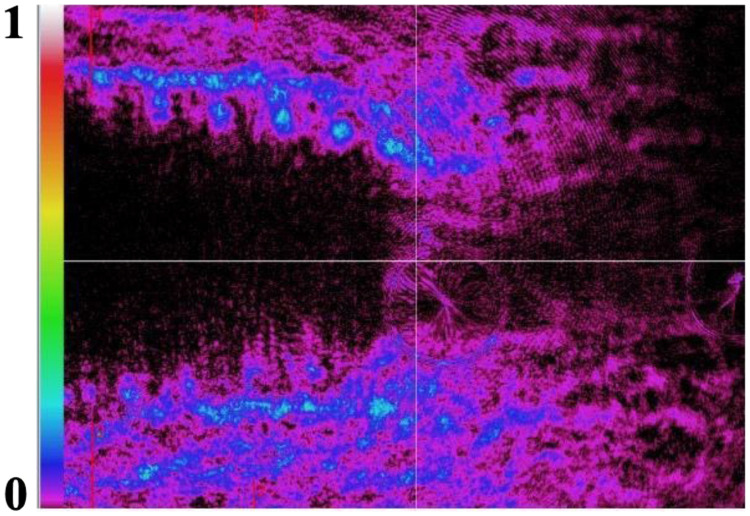
Example of the 3085 DI third replica polarized light optical intensity changes (ΔI) under static load from 0 to 150 N.

The results demonstrate the percentage change in polarized light intensity relative to the unloaded state, providing a quantitative basis for comparing the response of different samples to the applied load. The analysis was performed using three replicates of each DI. For visualization, an example of a subtracted 0 N polarized light image from the corresponding 150 N image is presented in Fig. 5.

**Fig. 6:**
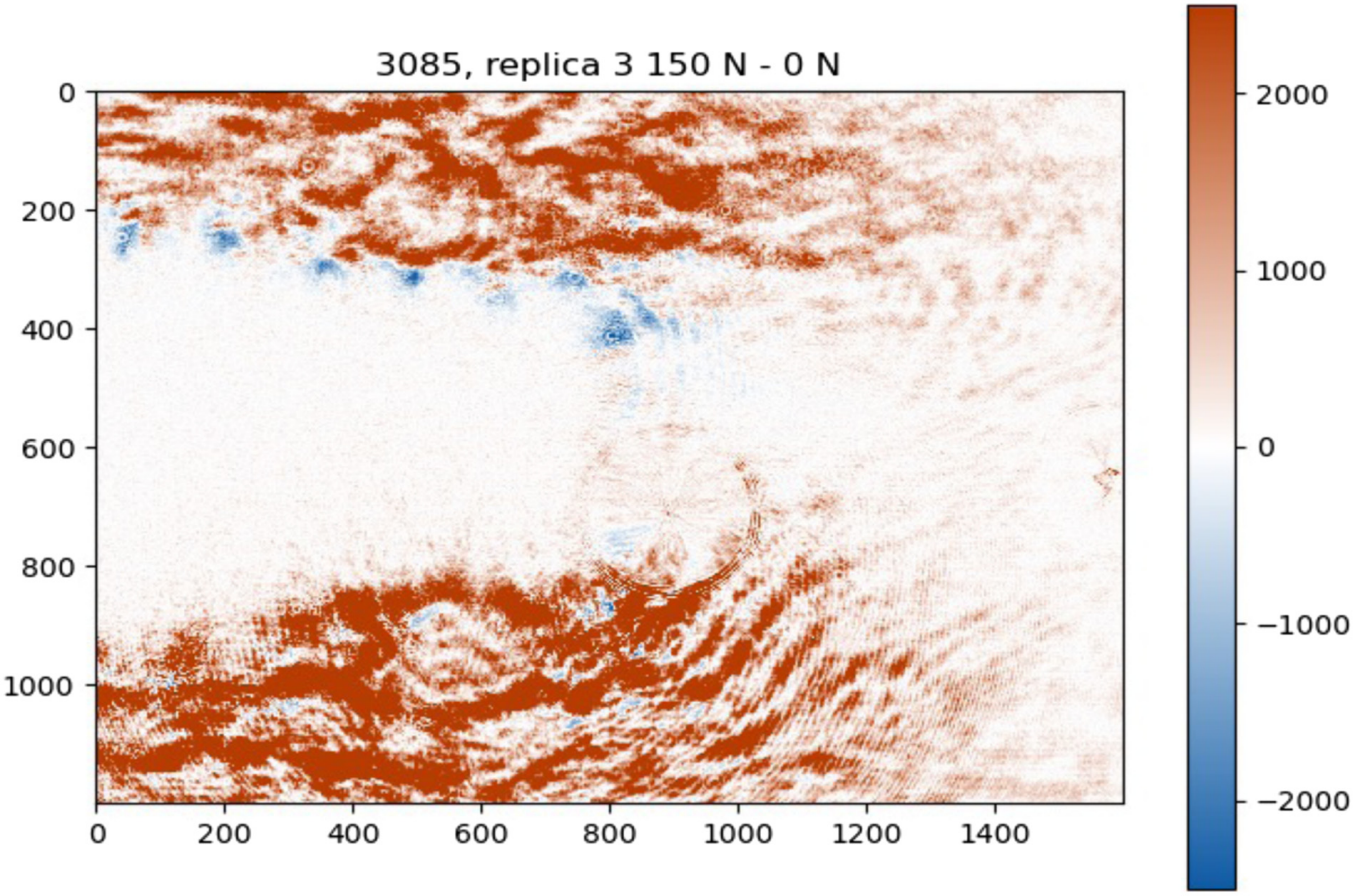
Area highlighted in yellow indicates the region used for calculations presented in different sections of the results. In this case, optical intensity changes (ΔI) under static load were analyzed across the entire image.

**Fig. 7:**
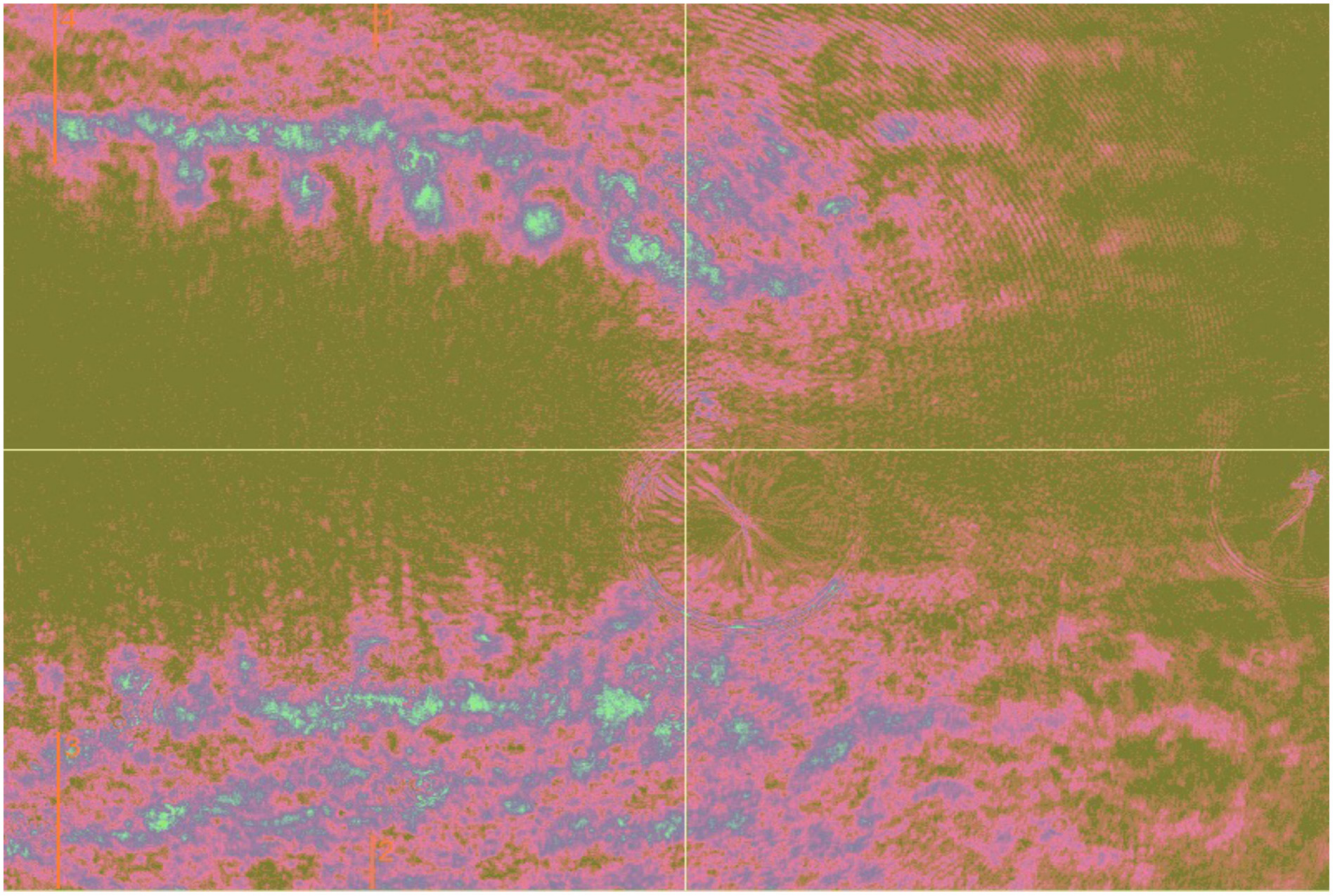
Area highlighted in yellow indicates the region used for calculations presented in different sections of the results. In this case, optical intensity changes (ΔI) under static load were analyzed only in the aperture area of BMM.

Two different parts of the results were obtained. Initially, the optical intensity changes (ΔI) under static load were calculated for the full-scale images containing 4 mm of DI and 3 mm of BMM under DI (Fig. 6). However, the limited camera resolution prevented us from expanding the image further. A wider DI would occupy more space in the image, leading to decreased intensity in the full-scale image, causing the results to be distorted. Therefore, the apical area of 3 mm of BMM under the DI was additionally selected for calculation (Fig. 7) to ensure more accurate results. Multiple regression analysis was conducted using Python 3.11.5 and the statsmodels 0.14.0 library to assess the relationship between DI width and length and load distribution in the BMM.

### 2.6 Theoretical Framework

Under crossed polarizers, the intensity transmitted through a stressed, birefringent specimen follows the stress-optic law: $I=I_{0}\;\sin^{2}(\frac{\Delta }{2}+\varphi_{0})$, where $\Delta =\frac{2\pi }{\lambda }Ct(\sigma_{1}-\sigma_{2})$ is the phase retardation, C is the stress–optic coefficient, t is the specimen thickness, λ is the wavelength, and ($\sigma_{1}-\sigma_{2}$) is the principal stress difference.

Because I(σ) is sinusoidal, we designed the optical and mechanical conditions (specimen thickness, 150 N load, analyzer orientation) so that the measurement field lay within a low-order, locally monotonic region, thereby avoiding multi-fringe patterns and phase wrapping. In this regime, the local linear approximation of I(σ) is routinely used for comparative assessments. Accordingly, we report ΔI as an optical response proxy for comparing implant diameter and length effects under identical conditions, rather than as absolute stress values.

### 2.7 Study of BMM

**Fig. 8:**
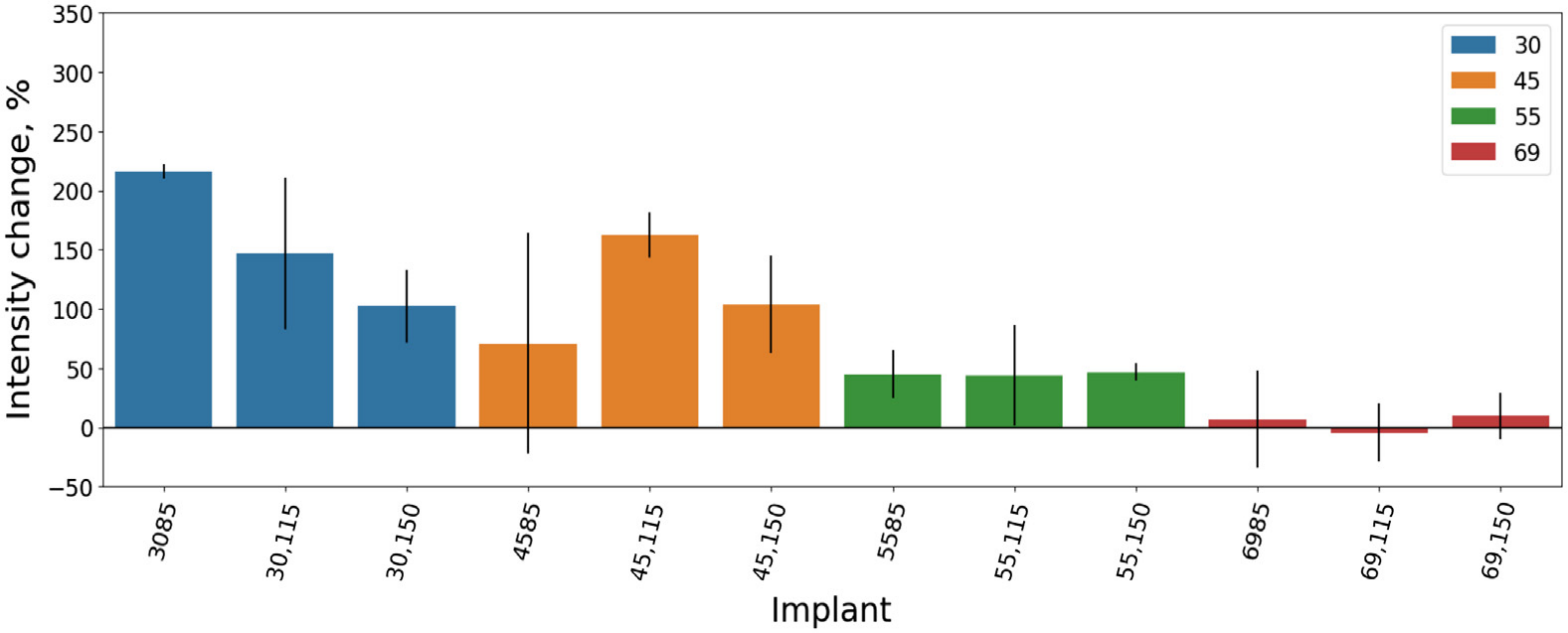
Results of the reproducibility of polarized light images for epoxy materials that match the BMM among three samples of each material, along with R-squared values: (a) FL210, (b) Liquidissima, and (c) Transparente.

Visual recordings were captured at two distinct positions on each of the three replicated workpieces—specifically at distances of 2 mm and 15 mm from the top, centered horizontally within each sample. The consistency between these recordings was assessed by measuring and comparing intensity variations at these defined measurement points. Analysis of the results indicated differing levels of consistency among the tested materials: BMM “Transparente” exhibited the highest consistency ($R^{2}=0.962$), followed by “FL210” ($R^{2}=0.868$) and “Liquidissima” ($R^{2}=0.737$), as presented in Fig. 8.

### 2.8 Study of DI Apical Area Optical Intensity Changes (ΔI) Under Static Load in Full-Scale Images

The study analyzed the optical intensity changes (ΔI) under static load in polarized light at the apical area of DI of varying diameters and lengths embedded in the selected BMM “Transparente” under identical optical and mechanical conditions. The results are summarized as average percentages of three replicas’ optical intensity changes (ΔI) under a static load of 150 N compared with the unloaded state 0 N.

After calculating polarized light changes using multiple regression. The dependence of DI diameter had a value of p<0.05, length p>0.05, const p<0.05, and adjusted R-squared = 0.594. Coefficients are also represented in Table 1.

**Table 1:** Optical intensity changes (ΔI) under static load of full-scale images depending on DI length and diameter p value, adj. R-squared and coefficient.

DI	Length	Diameter	Const
P value	$3.22\times 10^{-1}$	$2.74\times 10^{-8}$	$6.16\times 10^{-8}$
Adj. R-squared	0.594		
Coefficient	−0.30	−4.05	315.90

**Fig. 9:**
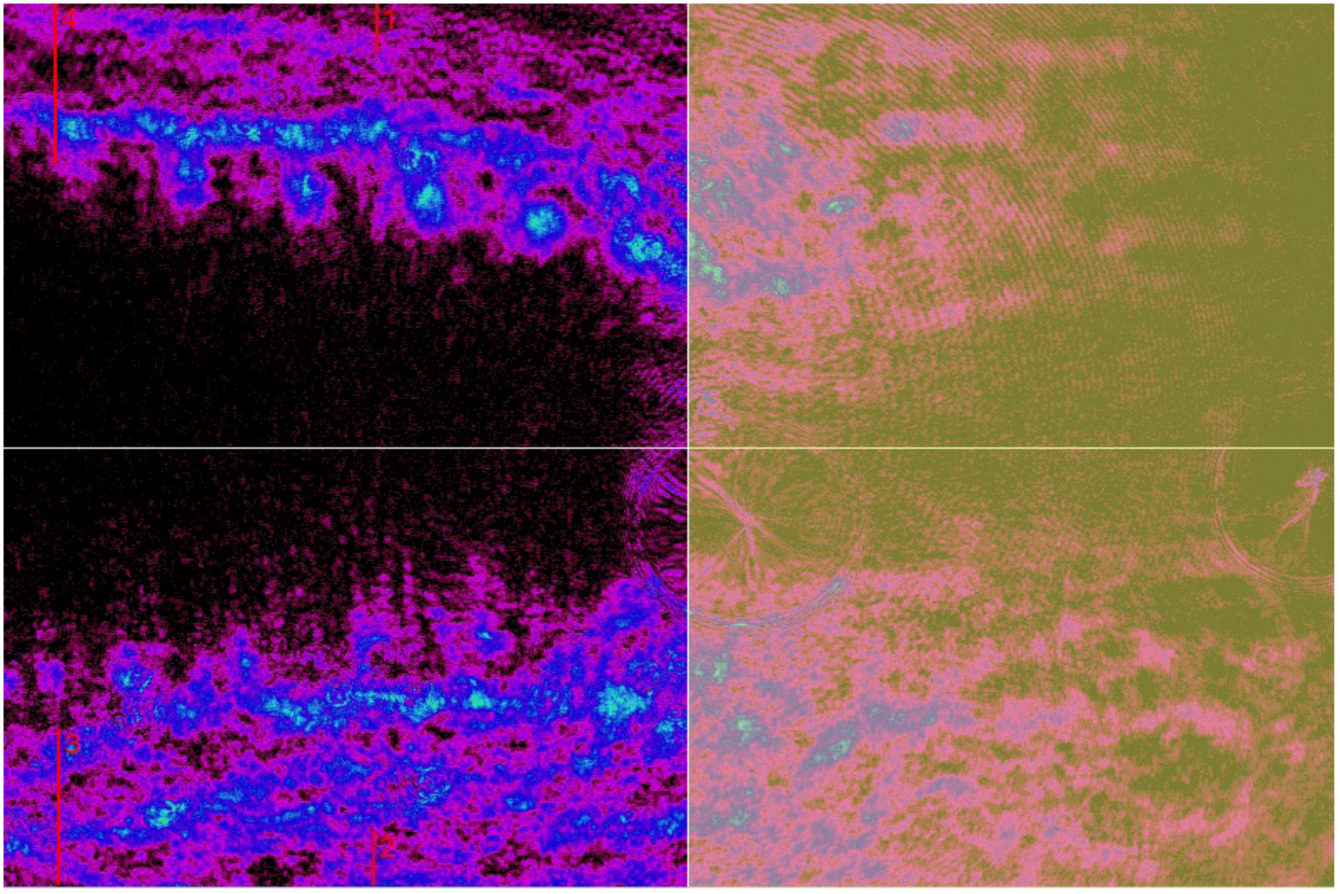
Polarized light average percentage of optical intensity changes (ΔI) under static load in BMM depending on DI length and diameter in full-scale images.

Longer DI transfers less static load to the nearby BMM has only been proven with a 3.0-mm diameter DI. However, 4.5-mm and 5.5-mm-diameter DI showed a bit different trend, where the 11.5-mm-length DI transfers the biggest part of static load to the BMM, and the lowest polarized light ΔI for 4.5-mm-diameter DI was observed with the 15-mm-length DI, and for the 5.5-mm-diameter DI—with the 8.5-mm-length DI. Meanwhile, the 6.9-mm-diameter and longest DI transferred the biggest part of the static load to the bone, and the 11.5-mm-length DI had the least polarized ΔI (Table 2, Fig. 9).

**Table 2:** Mean DI optical intensity changes (ΔI) under static load and SD of full-scale images.

DI	DI diameter (mm)	DI length (mm)	Optical intensity changes (ΔI) under static load mean (%) SD ±
3085	3.0	8.5	216.13 ± 5.98
30,115	3.0	11.5	147.09 ± 64.07
30,150	3.0	15.0	102.35 ± 31.02
4585	4.5	8.5	70.90 ± 93.22
45,115	4.5	11.5	162.24 ± 19.26
45,150	4.5	15.0	103.89 ± 41.26
5585	5.5	8.5	45.05 ± 20.34
55,115	5.5	11.5	43.70 ± 42.44
55,150	5.5	15.0	46.77 ± 7.48
6985	6.9	8.5	6.84 ± 41.02
69,115	6.9	11.5	−4.44 ± 24.51
69,150	6.9	15.0	9.72 ± 19.75

The difference in proportion between the highest and the lowest ΔI with a 3.0-mm-diameter DI was 113.78; with a 4.5-mm-diameter DI—58.35; with a 5.5-mm-diameter DI—19.12; and with a 6.9-mm-diameter DI—14.16 (Table 2, Fig. 9).

Wider DI demonstrated reduced polarized light ΔI, which means that it distributes static load transfer to the nearby BMM.^20^ DI with a 4.5-mm diameter (4585, 45,115, 45,150) and a 5.5-mm diameter (5585, 55,115, 55,150) transfers static load to the bone 1.21 and 2.98 times less than 3.0-mm-diameter (3085, 30115, 30150) DI. Meanwhile, DI with a 6.9-mm diameter (6985, 69,115, 69,150) showed 22.17 times less polarized light changes than 3.0-mm-diameter DI. Comparing 4.5-mm-diameter DI with 5.5-mm diameter showed 2.47 and 6.9-mm diameter showed 18.38 times less polarized light changes. Comparing the 5.5-mm-diameter DI with the 6.9-mm diameter showed 7.43 times less polarized light change (Table 2).

### 2.9 Study of DI Apical Area Optical Intensity Changes (ΔI) Under Static Load in the 3 mm Under the DI Images

The dependence of the DI diameter showed a significant effect, with a p<0.05, whereas the length showed no significant effect (p>0.05), and the constant was significant with a p<0.05. The adjusted R-squared value was 0.297. Coefficients are presented in Table 3.

**Table 3:** Intensity changes of 3 mm under the DI images depending on DI length and diameter p value, adj. R-squared and coefficient.

DI	Length	Diameter	Const
P value	$1.04\times 10^{-1}$	$6.93\times 10^{-4}$	$3.10\times 10^{-2}$
Adj. R-squared	0.297		
Coefficient	0.59	−2.45	119.84

**Fig. 10:**
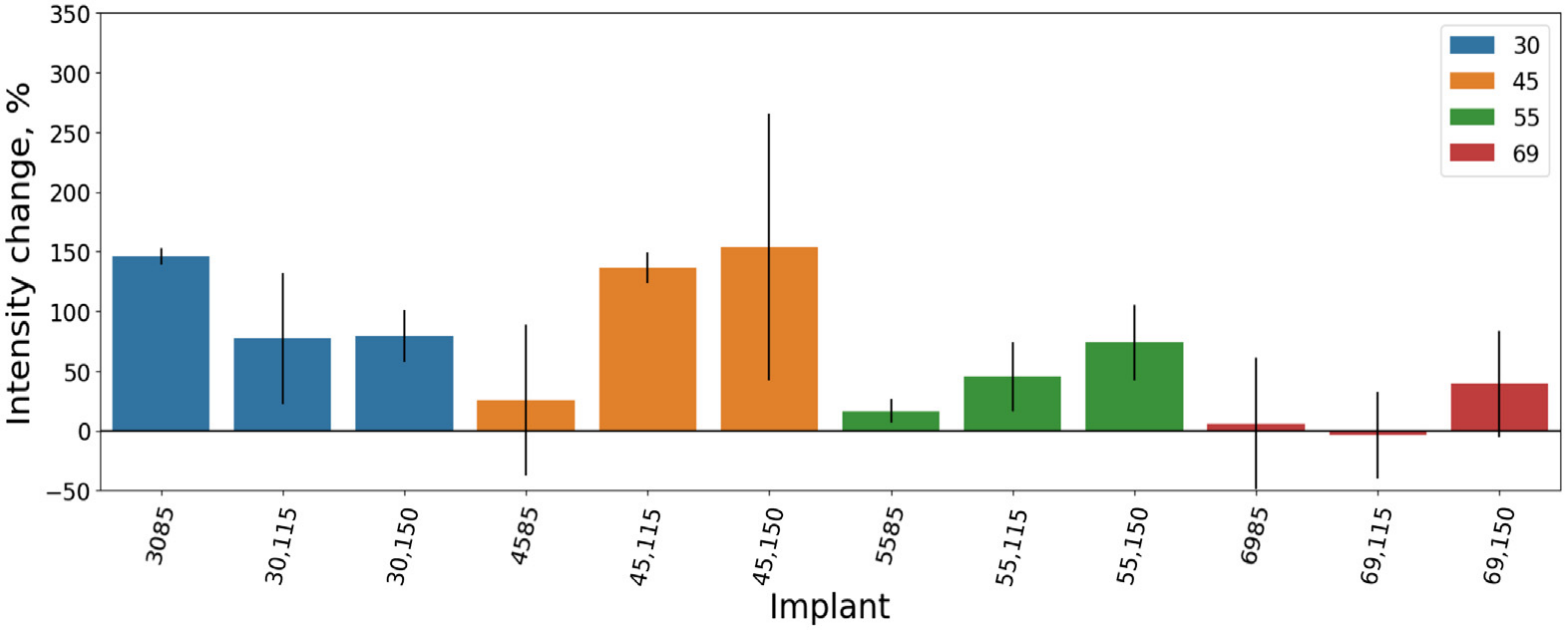
Polarized light average percentage of optical intensity changes (ΔI) under static load in BMM depending on DI length and diameter in 3 mm under the DI images.

For the 3.0-mm-diameter DI, with lengths of 11.5 mm and 15 mm, nearly identical intensity was observed, and the highest ΔI was noted with the 8.5-mm length DI. However, for the 4.5-mm and 5.5-mm-diameter DI, a different trend emerged, where the 15-mm length DI changed the greatest portion of the intensity, and the lowest polarized light ΔI occurred with the 8.5-mm length DI for both diameters. By contrast, the 6.9-mm diameter with the length of 15 mm DI had the biggest ΔI and the 11.5-mm length DI of the same diameter showed the least polarized light ΔI (Table 4, Fig. 10).

**Table 4:** Mean DI optical intensity changes (ΔI) under static load and SD of 3 mm under the DI images.

DI	DI diameter (mm)	DI length (mm)	Optical intensity changes (ΔI) under static load mean (%) SD ±
3085	3.0	8.5	145.90 ± 6.75
30,115	3.0	11.5	77.23 ± 54.74
30,150	3.0	15.0	79.34 ± 21.80
4585	4.5	8.5	25.80 ± 63.05
45,115	4.5	11.5	136.06 ± 12.96
45,150	4.5	15.0	153.88 ± 111.41
5585	5.5	8.5	16.42 ± 9.96
55,115	5.5	11.5	45.36 ± 29.06
55,150	5.5	15.0	73.85 ± 31.83
6985	6.9	8.5	6.03 ± 55.22
69,115	6.9	11.5	−3.92 ± 36.61
69,150	6.9	15.0	39.17 ± 44.69

The difference between the highest and lowest ΔI for each DI diameter was as follows: 68.67 for 3.0 mm, 128.08 for 4.5 mm, 57.43 for 5.5 mm, and 43.09 for 6.9 mm (Table 4).

Wider DI demonstrated reduced polarized light ΔI (except for the 4.5-mm diameter DI), which could indicate that these DI distribute the static load more effectively to the nearby BMM.^18^ DI with a 4.5-mm-diameter (average, 105.25) ΔI was nearly the same as the 3.0-mm-diameter DI (average, 100.82). The 5.5-mm-diameter DI showed a different trend, changing intensity 2.23 times less compared with the 3.0-mm-diameter DI. Meanwhile, the 6.9-mm-diameter DI exhibited 6.16 times less polarized light change than the 3.0-mm-diameter DI. Similar results were observed when comparing the 4.5-mm-diameter DI to the 5.5-mm-diameter DI, which showed 2.33 times less, and the 6.9-mm-diameter DI, which showed 6.43 times less polarized light change with the wider DI. Comparing the 5.5-mm-diameter DI with the 6.9-mm-diameter DI revealed 2.76 times less polarized light change (Table 4).

## 3 Discussion

Teeth transfer most of the received load to the periodontal ligament, whereas a DI does not have a periodontal ligament, and the pressure is directly transmitted to the bone.^21^ That is why this study investigates the effect of DI diameter and length on the intensity of polarized light changes in BMM under static loading conditions. The adjusted R-squared values were notably different when comparing the full-scale images (adj. R-squared = 0.594) to the 3 mm under DI images (adj. R-squared = 0.297). This discrepancy suggests that the full-scale images, which contain wider DI, occupy a larger area in the image, leading to decreased ΔI. This reduction in ΔI distorts the adjusted R-squared results and the diameter-dependent p value, which was statistically significant (p<0.05). The observed trend indicates that as the DI diameter increases, the ΔI decreases. However, the p value for calculating ΔI only in the 3 mm under DI images, where they are independent of the DI width, still showed statistical significance (p<0.05). Conversely, changes based on DI length were not statistically significant (p>0.05), suggesting that diameter could contribute more significantly to static load distribution than length. When comparing the ΔI between different diameters in two analyzed areas of the image, it is evident that the full-scale image analysis yields higher optical intensity changes ΔI under static load results. In the full-scale images, wider diameter DI generally exhibits a more even distribution of ΔI. However, there is an exception with the 4.5-mm-diameter DI, which, on average, shows less ΔI distribution than the 3.0-mm-diameter DI when analyzed in the 3 mm under DI images. Both analysis methods indicate that wider DI tends to show a smaller percentage difference between the maximum and minimum static load transfer to the surrounding BMM. The exception to this trend is again the 4.5-mm-diameter DI in the 3 mm under DI analysis. The hypothesis that the 4.5-mm-diameter DI did not align with the expected trend is likely due to the heterogeneous distribution of BMM. The discrepancies between the results can be attributed to the higher ΔI significance observed in most full-scale DI, except for the specific lengths of 4515, 55,115, and 5515. When comparing the length-related results between the full-scale and 3 mm under DI image analyses, a clear trend is not observed. The only consistent finding is with the full-scale 3.0-mm-diameter DI, where longer DI lengths correspond to reduced ΔI.

When comparing these study findings with others that investigate the influence of DI diameter and length on primary and secondary stability, an *in vivo* clinical study involving 88 DI placed in 63 patients demonstrated that DI diameter had a stronger influence than DI length, particularly when evaluated using RFA.^22^ Whereas others, using the same evaluation technique, RFA, tested 582 DIs placed in 272 patients and reported that length, in addition to diameter, contributes significantly to DI stability.^23^ However, evidence from other studies suggests that while increasing DI length initially improves the contact surface area with surrounding bone and enhances primary stability, this locally monotonic relationship plateaus at DI lengths beyond ∼12  mm.^24^ This indicates that simply increasing length beyond this point does not contribute significantly to further stability gains. Supporting this, an *in vitro* study demonstrated that higher implant stability quotient (ISQ) values were achieved with longer, 10-mm-length DI, suggesting improved primary stability at this length. Nevertheless, primary stability was found to be similar between 4-mm and 6-mm length DIs.^25^ These kinds of studies are also commonly conducted in computer simulations using FEA, and most of them have found that DI diameter and length have an influence on DI stability,^26^ but diameter dependence is greater.^27^^,^^28^

Some studies utilizing photoelastic analysis and embedding DI in acrylic resin (Young’s modulus ∼3.5  GPa and compressive strength 85 to 110 MPa^29^ reveal a trend indicating that both DI length and diameter influence static load distribution.^16^ One study employed photoelastic III epoxy resin (Polipox) (Young’s modulus 0.0045 GPa),^30^ and it was observed that the 11-mm length DI distributed stress more favorably than the 6 mm DI.^31^ In addition, another study demonstrated comparable findings using DI cast into Araldite GY279BR photoelastic resin (Young’s modulus 1.3 GPa and compressive strength 65 MPa,^32^ showing that the 15-mm length DI distributed stress more favorably than the 10 mm DI.^33^ In other investigations, PL-2 epoxy resin (Young’s modulus 0.21 GPa) and composite photoelastic models were utilized, with findings indicating that larger-diameter DIs exhibited better stress distribution than narrower DIs.^34^^,^^35^ Among the materials used, Araldite GY279BR photoelastic resin and acrylic resin exhibit mechanical properties most similar to the “Transparente” epoxy resin, particularly when comparing Young’s modulus. Nevertheless, due to methodological differences, these studies cannot be considered fully comparable.

Digital photoelasticity has emerged as a valuable tool for analyzing stress distribution in implant dentistry, particularly in full-arch rehabilitations such as the all-on-four concept. It facilitates the visualization of stress intensity changes associated with distal implant inclination, which in some studies are evaluated in anatomically relevant mandibular models.^18^^,^^36^ Ramesh later refined this methodology in a subsequent study, further highlighting the growing application of digital photoelastic techniques in dentistry.^37^ Beyond implantology, photoelasticity has also been employed in orthodontics, operative dentistry, and occlusion studies.^38^ Comparative analyses of DIC and photoelasticity indicate that while both methods reveal similar qualitative stress patterns, photoelasticity is particularly sensitive to minor stress variations, whereas DIC offers quantitative data across a wider range of materials.^19^ Moreover, the integration of FEA with photoelasticity enhances the reliability of stress distribution assessments and provides deeper insight into the effects of implant length and thread pitch on biomechanical performance.^39^

Recent developments in photoelasticity also highlight the potential of emerging computational approaches. Hybrid phase-shifting methods have been shown to improve efficiency in extracting both isoclinic and isochromatic data from fewer measurements.^40^ Deep learning techniques such as PhotoelastNet can generate full-field stress maps from a single color fringe image with high accuracy,^41^ whereas neural network–based fringe demodulation models, such as FringeNet, offer improved reliability in processing isochromatic patterns.^42^ More recently, neural implicit models such as NeST have been proposed to enable 3D stress reconstruction from photoelasticity data.^43^ Although these approaches are not yet commonly applied in DI biomechanics, acknowledging them provides a forward-looking perspective and places the present study within the broader methodological progress of experimental stress analysis.

The finding that wider DI reduces polarized light ΔI under a vertical static load of 150 N has critical implications for clinical practice. In scenarios where patients exhibit sufficient horizontal alveolar bone but inadequate vertical bone height, clinicians may consider choosing shorter yet wider DI to avoid bone augmentation procedures or the risk of perforating anatomical structures. This approach could simplify treatment protocols and reduce the complexity and morbidity associated with bone grafting.

In addition, this study does not fully replicate physiological conditions, such as those present in the natural mastication process. Factors such as the influence of facial muscles and the use of a straight abutment instead of a dental crown may affect the results and limit their direct applicability to clinical scenarios. Nevertheless, the selected epoxy resin (“Transparente”) exhibited a reported density of $\;1100\;\mathrm{kg}/m^{3}$, closely matching the mean density of mandibular trabecular bone with marrow *in situ*, which is $1140\;\mathrm{kg}/m^{3}$.^44^ For reference, calculated densities in human jawbone regions vary considerably: anterior maxillary bone exhibits a mean density of $∼790\;\mathrm{kg}/m^{3}$, posterior maxillary bone $∼570\;\mathrm{kg}/m^{3}$, anterior mandibular bone $∼1180\;\mathrm{kg}/m^{3}$, and posterior mandibular bone $∼1190\;\mathrm{kg}/m^{3}$.^45^ The epoxy resin exhibits a Young’s modulus of ∼2.5  GPa and a compressive strength of about 80 MPa, whereas jawbone regions vary considerably: anterior maxillary bone—14.5 GPa; posterior maxillary bone—15.3 GPa; anterior mandibular bone—16.8 GPa; posterior mandibular bone—19.7 GPa; and a compressive strength between 100 and 230 MPa.^45^^–^^47^ Although the BMM does not perfectly replicate the stiffness and strength of the natural bone, it provides a reasonable approximation of mandibular trabecular bone density. Although the lower stiffness of epoxy resin may result in underestimation of absolute stress magnitudes, the consistency of the testing conditions across all samples ensures that the observed comparative trends between different DI designs remain valid. Optical clarity and reproducibility under polarized light were prioritized in material selection to ensure accurate stress visualization, which was essential for the methodology applied.

**Fig. 11:**
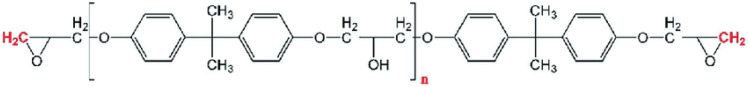
General chemical structure of DGEBA.^48^

The chemical structure of the “Transparente” epoxy resin also contributed significantly to its mechanical and optical properties. The resin is based on a diglycidyl ether of bisphenol A (DGEBA) backbone, a widely used compound in optically transparent, mechanically stable epoxy systems. Upon curing, the terminal epoxide groups react with hardeners (commonly amines or anhydrides), forming a highly cross-linked thermosetting polymer network. This crosslinked architecture contributes to the material’s rigidity and compressive strength while limiting its ductility. However, unlike the anisotropic, hierarchical structure of cortical bone, DGEBA-based epoxy exhibits a homogeneous and isotropic internal configuration. This structural simplicity results in uniform stress distribution patterns, making the material suitable for comparative optical stress analysis but not ideal for replicating the full biomechanical behavior of the human bone. The general chemical structure of DGEBA is presented below (Fig. 11):

Although this molecular architecture does not fully replicate the elastic and failure behavior of bone, its consistency and clarity under optical loading conditions make it a viable material for photoelastic studies focused on relative stress distribution.

The standard deviation (SD) observed in some of the DI was notable, which can be attributed to variability in the polarized light images generated by the replicas. Each time polarized light passed through the replica workpieces, different recordings were produced. This variability indicates that the polarization was scattered slightly differently each time. The primary reason for generating different polarized light recordings in the same replicas is likely due to variations in the molecular arrangement within the BMM, which directly affects the material’s homogeneity.^49^ Several factors could contribute to this variability: temperature during the curing process, air bubbles present in the BMM, despite efforts to use a vacuum chamber, humidity during casting, which could influence the material’s curing process, and material properties of the BMM itself, which may have inconsistencies in its molecular structure.^50^ Comparing full-scale to 3 mm under DI images, the ΔI SD is more notable in 3 mm under DI images. This could be because DI threads visible in the recorded image are more repetitive in different replica images compared to only visible BMM in 3 mm under DI images. To improve the consistency and reliability of the results, the same testing method should be applied to more homogeneous yet transparent materials. Using advanced materials with better-controlled properties would likely reduce variability and enhance the reproducibility of findings.

In addition, although the present study focused on evaluating relative static load distribution using photoelastic analysis, further validation through complementary testing would strengthen the methodology. Future investigations should consider integrating additional *in vitro* testing on extracted bone samples or bone-like composite substrates, including push-out tests, micro-CT analysis, or nanoindentation to comprehensively assess implant–bone interface behavior.

Measuring the elastic modulus and compressive strength of the cured BMM under axial and lateral loading would also provide a more accurate representation of its mechanical performance.

Regarding the experimental setup, the resolution of the CCD camera used in capturing light ΔI was 1200×2400 with a pixel size of 4.4  μm. Image fidelity remains a factor influencing quantitative interpretation. However, such a resolution should provide a sufficient field of view and accuracy of localized measurements.

In future studies, combining the experimental method with FEA could enable simulation of more complex loading scenarios, including oblique forces, cyclic loading patterns, and variations in bone density. This hybrid experimental-computational approach could significantly enhance the predictive modeling of DI behavior under diverse clinical conditions.

## 4 Conclusion

The testing method utilized in this study effectively assessed the relationship between changes in BMM intensity and the diameter of DI. Analysis showed a significant correlation, with a p value<0.05 in both full-scale and 3 mm under DI images, suggesting that DI diameter plays a key role in BMM optical intensity changes (ΔI) under static load. On the contrary, no significant dependence was found between BMM ΔI and DI length, as evidenced by a p value>0.05 across both result types. This indicates that within the tested range, DI length does not substantially affect the ΔI in BMM.

In addition, this approach is cost-effective and requires minimal equipment, making it practical for use in both research and clinical environments. Its simplicity and accuracy in assessing the relationship between DI dimensions and BMM response highlight its potential for widespread application. The method offers an accessible way to test various DI configurations in BMM, making it a useful tool for experimental purposes.

In summary, this testing technique provides valuable insights into the mechanical interactions between DI and surrounding BMM, with particular focus on the effect of DI diameter. Future investigations may explore the influence of other DI factors, such as material properties or surface characteristics, to further deepen the understanding of DI behavior within BMM.

## Data Availability

The dataset supporting the findings of this study has been deposited in Figshare and is openly available at: https://doi.org/10.6084/m9.figshare.29549600. It includes all raw and processed data used in the photoelastic analysis of dental implants. No custom code was created or used in this study.
